# Combination Therapy Using Chelating Agent and Zinc for Wilson’s Disease

**DOI:** 10.1007/s40846-015-0087-7

**Published:** 2015-11-19

**Authors:** Jui-Chi Chen, Cheng-Hung Chuang, Jing-Doo Wang, Chi-Wei Wang

**Affiliations:** Department of Computer Science and Information Engineering, Asia University, Taichung, 41354 Taiwan; Department of Medical Research, China Medical University Hospital, Taichung, 40402 Taiwan; Department of Biomedical Informatics, Asia University, Taichung, 41354 Taiwan; Department of Internal Medicine, Ben Tang Cheng Ching Hospital, Taichung, 41364 Taiwan

**Keywords:** Biomedical informatics, Wilson’s disease, Combination therapy, Effectiveness, Safety

## Abstract

There is no clear international consensus regarding the optimal medication therapy for treating Wilson’s disease (WD). This study systematically reviews the effectiveness of various medication therapies in common use, specifically focusing on preliminary findings concerning the combination of a chelating agent and zinc. A systematic PubMed search was executed to locate original studies on the effectiveness of commonly used medications for WD published between January 1989 and August 2014. The results were used to conduct a systematic review of studies on combination therapies. A total of 17 combination therapy studies involving 1056 patients were reviewed. These were analyzed in terms of data on effectiveness, adverse effects, and mortality. Results from a pooled analysis indicate that combination therapies for hepatic patients were significantly less effective than the same therapies for neurological manifestations (47.1 vs. 78.6 %; pooled relative risk ratio (RR): 0.63, 95 % confidence interval CI 0.43–0.94; *p* = 0.02). Data from a subgroup analysis show that the combination therapy of penicillamine plus zinc sulfate resulted in a significantly higher mortality rate compared to all other combination therapy types (16.3 vs. 4.7 %; RR: 3.51, 95 % CI 1.54–8.00; *p* < 0.001). The use of combination therapies involving zinc and a chelator should be carefully monitored with close clinical observations and frequent biochemical tests, especially for WD patients with hepatic manifestations.

## Introduction

Wilson’s disease (WD) (also known as hepatolenticular degeneration, or HLD) is a rare inherited autosomal recessive disorder associated with mutations in the adenosine triphosphatase 7B (ATP7B) gene [1–18] and characterized by copper metabolic abnormalities [19, 20]. Excessive copper accumulation can result in toxicity and damage to the brain, liver, kidney, and other tissues. WD has a broad spectrum of clinical presentations, with hepatic and neurological symptoms considered the main features [21]. While liver transplants (LTs) and gene therapies are provided to a small number of WD patients, the large majority require lifelong medication to control the absorption and storage of copper in their bodies. The most commonly used drugs are penicillamine (DPA) [22], trientine (TETA) [23], zinc salts (Zn) [24, 25], and tetrathiomolybdate (TM; an experimental therapy that is not yet commercially available) [26, 27]. The goal of medication is to prevent, stabilize, or reverse copper overload and WD symptoms [28].

The European Association for the Study of the Liver (EASL) [19] and the American Association for the Study of Liver Diseases (AASLD) [20] announced their respective clinical practice guidelines for WD in 2012 and 2008, but no clear international consensus exists regarding an optimal medication therapy. One reason is the diversity of WD genotypes and phenotypes, which makes it difficult to determine differences in drug effectiveness. Another challenge is the small number of known cases, with a worldwide prevalence of 1/30,000 [18]. This makes it difficult to conduct large-scale cohort randomized clinical trials. Our motivations were to collect and compile available data from past studies on the effectiveness and safety of commonly used WD medications, and to review original studies found in the PubMed database.

In combination therapies for WD, zinc and a chelating agent (chelator) are utilized to block copper uptake and to eliminate excess copper [16]. The two medications must be taken at least 1 hour apart in order to mitigate zinc chelation [14]. They are still considered controversial, with few rigorously designed studies and little in the way of safety data [29]. The most frequently cited studies that suggest favorable outcomes for combination therapy using DPA plus zinc or TETA plus zinc [29] are those by Dhawan et al. [30], Askari et al. [31], and Santos Silva et al. [32]. However, some earlier studies [33, 34] reported no advantages for the DPA-zinc combination. Both EASL [19] and AASLD [20] assert that considerably more research is required to determine whether combination therapy using a chelator plus zinc has advantages for WD patients. Therefore, our primary goal was to verify whether combination therapies are effective and safe at statistically significant levels for patients with different clinical presentations.

## Materials and Methods

### Search Strategy and Study Selection

We performed a systematic search of the National Center for Biotechnology Information’s PubMed database [35] for original WD treatment studies published between January 1989 and August 2014. Search keywords were “Wilson’s disease” or its synonyms (including “HLD”), and at least one of the most commonly used drugs: “penicillamine,” “trientine,” “zinc,” “tetrathiomolybdate”, and their brand names, acronyms, abbreviations, and synonyms. Inclusion criteria included prospective, retrospective, randomized, and non-randomized controlled studies with human subjects published as full articles written in English or Chinese. Exclusion criteria included animal studies, case reports or case series, reviews, letters, short papers, editorials, metal metabolism or pharmacological research, diagnostic or other testing studies, liver transplants or other non-medication treatments, duplicate reports, and insufficient data. Date of last search: September 1, 2014.

### Definition of WD Phenotypes

Four phenotype presentation categories were noted: neurological, hepatic, mixed, and asymptomatic. Following the lead of Ferenci et al. [36], patients with neurological and/or psychiatric symptoms at diagnosis were classified as neurological. The definition of hepatic presentation required the exclusion of neurological symptoms noted during a detailed examination at the time of diagnosis [36]. Pure hematological abnormalities such as Coombs negative hemolytic anemia were classified as hepatic. Asymptomatic manifestations included asymptomatic and pre-symptomatic presentation. Finally, patients with other miscellaneous symptoms (e.g., renal dysfunction and bone deformities) were placed in a mixed presentation category with patients showing simultaneous hepatic and neuropsychiatric symptoms.

### Treatment Effect Definition

To maintain consistency, we used a comparative unit called “treatment block” (TB) [16] to calculate the frequencies of adverse effects and treatment effectiveness for patients during specific time durations. One TB equaled the duration of one therapy up to the time that a medication was changed, or until the end of the follow-up period [16]. For our purposes, a TB was considered effective when the author of a research paper used terms such as “effective,” “efficacious,” “successful,” “improved,” “stable,” “normal,” “biochemically improved,” “responsive,” “non-progressive”, or their synonyms. The list of terms indicating non-effectiveness included “ineffective,” “inefficacious,” “failed,” “deteriorated,” “worsened,” “degenerated,” “abnormal,” “severe side effects,” “LT,” “dead,” “treatment failure,” “stationary,” “unchanged,” “clinical suspicion,” “progressive,” “non-responsive”, or their synonyms.

### Data Extraction

To select studies according to our inclusion and exclusion criteria, two authors initially screened the entry information, titles, and abstracts of all retrieved records. Next, full texts were scanned to determine conformance with the criteria. Two authors independently extracted data and outcomes using a standardized form. All disagreements were discussed with a third author. Studies were included and data extracted in cases where a consensus was achieved. Other extracted information included first author, country, publication year, number of patients, patient gender ratio, patient phenotype ratio, adverse effects, and mortality (liver transplant and deceased counts).

### Statistical Analyses

All analyses were performed using Cochrane RevMan 5.3 and SPSS Statistics 22.0. Pooled relative risk ratios (RRs) and 95 % confidence intervals (CIs) were calculated from the original study data using the Mantel–Haenszel method with a random-effects model. A fixed-effects model was selected for cases with low heterogeneity (*I*^2^ < 30 %). The Mantel–Haenszel method generates estimates of associations between exposures and outcomes after accounting for confounding effects. We stratified the data into two or more confounding factor levels before computing pooled RRs across the strata. Note that the random-effects model has a stricter assumption than the fixed-effects model. We used the random-effects model to achieve conservative RR and CI estimates.

The Mantel–Haenszel equation for an RR is:

1 $${\text{RR }} = \, {{\sum\limits_{j = 1}^{m} {\frac{{a_{j} (c_{j} + d_{j} )}}{{n_{j} }}} } \mathord{\left/ {\vphantom {{\sum\limits_{j = 1}^{m} {\frac{{a_{j} (c_{j} + d_{j} )}}{{n_{j} }}} } {\sum\limits_{j = 1}^{m} {\frac{{c_{j} (a_{j} + b_{j} )}}{{n_{j} }}} }}} \right. \kern-0pt} {\sum\limits_{j = 1}^{m} {\frac{{c_{j} (a_{j} + b_{j} )}}{{n_{j} }}} }}$$

where *a*_*j*_, *b*_*j*_, *c*_*j*_, and *d*_*j*_ are the numbers of patients in each cell of a two-by-two table in the *j*-th stratum of the confounding variable, *n*_*j*_ represents the number of patients in the *j*-th stratum, and *m* is the total number of strata.

Correlations and associations between discrete values of nominal data variables from different treatment groups were evaluated using a Pearson Chi square (χ^2^) test and Phi or Cramer’s V measures. We used Chi square tests to determine the likelihood of independence between effectiveness/safety and different medications/phenotypes. A rejected null hypothesis suggested some degree of correlation between the two variables. To obtain measures of association between those variables, Phi or Cramer’s V values were calculated using a value of between 0 and 1. A measure of association achieved a maximum numerical value of 1 when the two variables had a perfect relationship with each other, and a value of 0 when there was no relationship. After the observed measure of association values had been calculated, if the measure was significantly different from 0, it was viewed as showing a significant relationship between the two variables. A *p* value of <0.05 was considered statistically significant.

## Results

### Included Literature

**Fig. 1:**
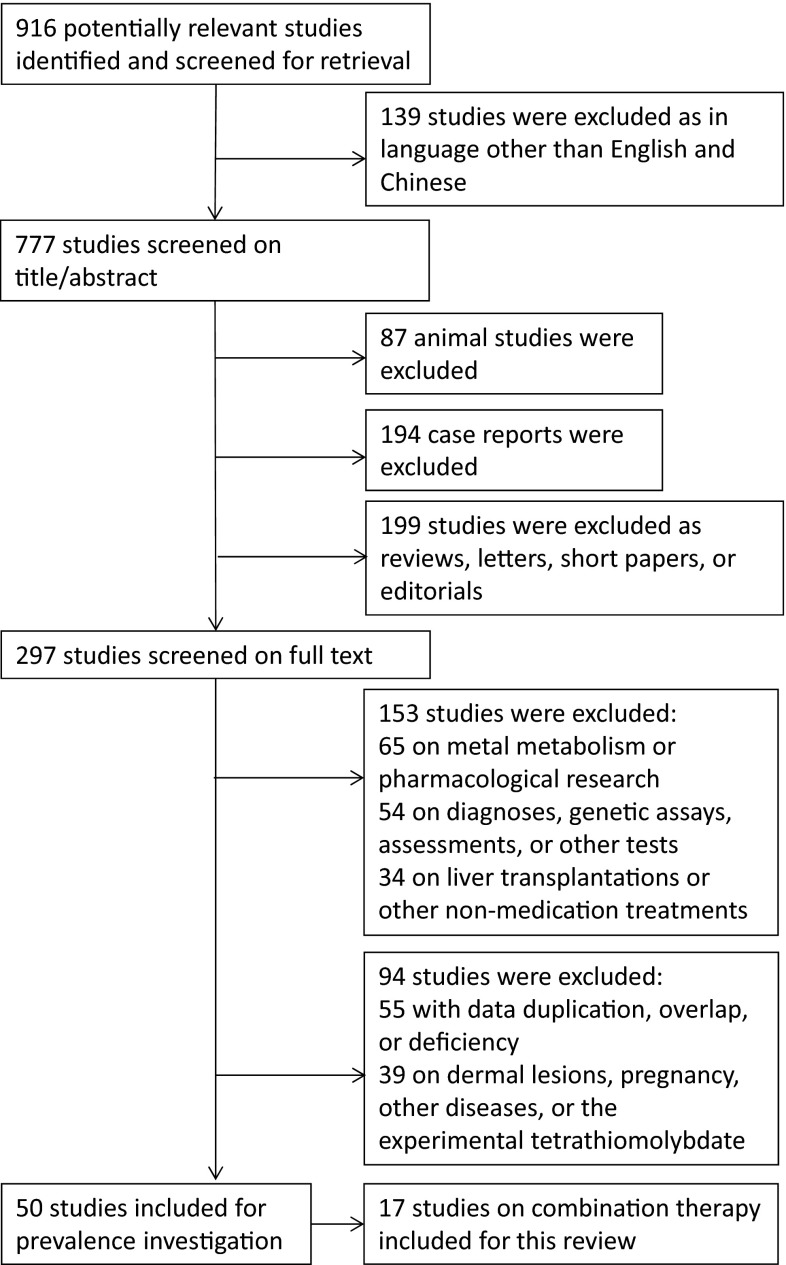
Flow chart of included and excluded studies for this review

A total of 916 hits were screened, 139 of which were excluded because they were written in languages other than English or Chinese. Another 480 studies were excluded during the secondary selection process of reading the retrieved titles and abstracts, and 245 were excluded during the tertiary step of scanning the full texts of potentially eligible studies. A total of 50 studies were included for prevalence investigation, and of these, 17 described outcomes from combination therapies and were therefore accepted for this review [1–17]. The study selection procedure is summarized in Fig. 1, characteristics of the 17 studies are shown in Table 1, and mean follow-up times and outcomes regarding the effectiveness of combination therapies in each study are shown in Table 2. As shown, the papers in the final sample discussed seven combinations of a chelator and a zinc salt: (a) DPA + Zn sulfate, (b) DPA + unknown or another Zn salt (e.g., zinc gluconate), (c) TETA + Zn sulfate, (d) TETA + Zn acetate, (e) TETA + unknown or another Zn salt, and (f) unknown chelator DPA or TETA + any Zn salt.

**Table 1:** Characteristics of 17 included studies on combination therapies

PMID	Study	Country	Mean age (years)	No. of patients	Gender		Phenotype				Data collected	
					Male	Female	Nue.	Hep.	Mixed	Asy.	From	To
23011036	Sini et al. [1]	Italy	23.0	60	19	41	0	38	22	0	1981	2011
22055589	El-Karaksy et al. [2]	Egypt	10.3	54	31	23	5	33	3	13	1996	2009
22355993	Noureen and Rana [3]	Pakistan	9.1	50	34	16	46	0	4	0	2005	2008
21682854	Abdel et al. [4]	Egypt	10.0	77	43	34	6	35	9	27	1992	2009
17709362	Sinha et al. [5]	India	14.4	50	30	20	39	3	8	0	1999	2002
14759316	Li et al. [6]	China	10.0	21	9	12	6	9	6	0	1990	1998
11837754	Sinha et al. [7]	India	13.3	49	38	11	27	0	22	0	1991	2000
10869138	Kalra et al. [8]	India	7.2	25	14	11	5	7	9	4	1986	1997
10745386	Yüce et al. [9]	Turkey	9.0	34	19	15	4	30	0	0	1980	1998
9193846	Schumacher et al. [10]	Germany	27.0	13	5	8	4	7	2	0	1988	1995
8076990	Gill et al. [11]	India	19.6	11	7	4	0	11	0	0	–	–
11819363	Ren et al. [12]	China	19.0	120	65	55	–	–	–	–	1994	1997
16606763	Brewer et al. [13]	USA	–	23	–	–	23	0	0	0	–	–
17460493	Arnon et al. [14]	USA	12.5	22	11	11	0	15	2	0	1998	2006
20958917	Bruha et al. [15]	Czech	38.5	117	59	58	21	51	34	11	1965	2008
21185835	Weiss et al. [16]	Germany and Austria	17.5	288	123	165	60	157	39	32	1954	2008
24661374	Ranucci et al. [17]	Italy	6.0	42	30	12	0	38	4	0	1984	2012
Total			17.6	1056	537	496	246	434	164	87		
Percentage (%)			(mean)		52.0	48.0	26.4	46.6	17.6	9.3		

*PMID* PubMed literature ID, *Neu.* neurological, *Hep.* hepatic, *Asy*. asymptomatic or presymptomatic, – not available

**Table 2:** Outcomes of 17 included studies on combination therapy effectiveness for WD

PMID	Mean follow-up	Comb. type	Total dose (mg/d) used		Comb.TB#	Stratified by phenotype								Overall effectiveness	
						Neurological		Hepatic		Mixed		Asymptomatic			
			Chelator	Zn		NE+	NE−	HE+	HE−	ME+	ME−	AE+	AE−	E+	E−
23011036	25.0	a	600–1200	150–300	13			6	2	1	4			7	6
22055589	2.0	a	40/kg	75–150	41	4	1	10	23	2	1			16	25
22355993	3.0	a	10–30/kg	50–100	50	46	4							46	4
21682854	4.9	a	18/kg(mean)	50–150	58	2	2	11	16	3	5	11	8	27	31
17709362	4.1	a	–	–	50									36	14
14759316	–	a	10–30/kg	67.5	18	9	2	13	5					13	5
11837754	3.8	a	500–1000	100	18									5	13
10869138	–	a	8–30/kg	50	20									13	7
10745386	9.0	a	20/kg	68.2	26	4	0	11	11					15	11
9193846	–	a	–	–	6	0	4	0	2					0	6
8076990	–	a	500–1000	100–150	6			4	2					4	2
	–	c	500	100	3			2	1					2	1
11819363	2.8	b	1000	80	60									35	25
16606763	1.0	e	1000	100–150	23	16	7							16	7
17460493	2.5	d	500–1000	50–100	2			0	2					0	2
20958917	15.1	b	600–1200	150	2	0	2							0	2
21185835	17.1	f	–	–	30									22	8
24661374	12.0	b	750–1500	50–150	11									7	4
Mean/Total	10.6				437	81	22	57	64	6	10	11	8	264	173
Percentage (%)	years					78.6	21.4	47.1	52.9	37.5	62.5	57.9	42.1	60.4	39.6

Comb. type (combination therapy type): *a* DPA + Zn sulfate, *b* DPA + Zn, *c* TETA + Zn sulfate, *d* TETA + Zn acetate, *e* TETA + Zn, *f* chelator + Zn; *Comb. TB#* number of treatment blocks on combination therapy; *Zn* zinc *E+* effective *E−* ineffective; – not available

### Prevalence Investigation

Of the 2954 WD patients mentioned in 45 of the 50 studies included for the prevalence investigation, 1357 (45.9 %) were female (95 % CI 44.1–47.7 %). Pooled mean age at diagnosis as mentioned in 47 of the same 50 papers was 18.7 years, ranging from 6 to 40 years. For our phenotype prevalence investigation, of the 2988 patients mentioned in 47 of the 50 included studies, those with neurological, hepatic, mixed, or asymptomatic presentations numbered 1058 (35.4 %), 1242 (41.6 %), 341 (11.4 %), and 347 (11.6 %), respectively. The number of hepatic patients was approximately 1.2 times that of neurological patients. When combined with the mixed phenotype, the total number of hepatic patients (i.e., at least one liver-related symptom) was 1583 (52.9 % of the total patient sample).

### Effectiveness

**Fig. 2:**
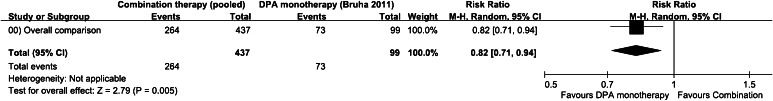
Forest plot of 17 included studies measuring relative risk of pooled effectiveness following combination therapy compared to DPA monotherapy [15]

**Fig. 3:**
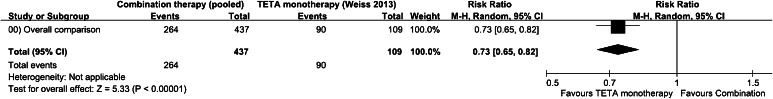
Forest plot of 17 included studies measuring relative risk of pooled effectiveness following combination therapy compared to TETA monotherapy [37]

**Fig. 4:**
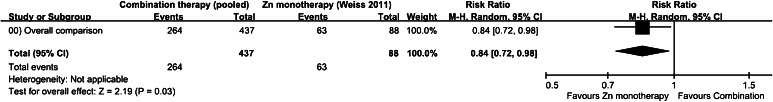
Forest plot of 17 included studies measuring relative risk of pooled effectiveness following combination therapy compared to Zn monotherapy [16]

Of the 437 pooled TBs shown in Table 2, 264 responded positively to a combination therapy, for an overall effectiveness rate of 60.4 % (95 % CI 55.8–65.0 %), lower than the rates reported by Bruha et al. for DPA monotherapy (73/99, or 73.7 % (95 % CI 65.1–82.4 %)) [15], Weiss et al. for TETA monotherapy (90/109, or 82.6 % (95 % CI 75.4–89.7 %)) [37], and Weiss et al. for zinc monotherapy (63/88, or 71.6 % (95 % CI 62.2–81.0 %)) [16]. As shown in Table 3, results from our inter-study analysis indicate significant differences in effectiveness rates between combination therapies and the three monotherapies: an RR of 0.82 for DPA [15] (95 % CI 0.71–0.94, Fig. 2), an RR of 0.73 for TETA [37] (95 % CI 0.65–0.82, Fig. 3), and an RR of 0.84 for Zn [16] (95 % CI 0.72–0.98, Fig. 4). In this part of our study, we used the number of effective TBs as the number of events.

**Table 3:** Effectiveness rates and RRs between combination therapies and three monotherapies as found in literature

	No. of TBs	Effective TBs	Effective rate (%)	Effective 95 % CI (%)	RR between combination therapies and others
On combination therapies (pooled)	437	264	60.4	55.8–65.0	–
On DPA monotherapy [15]	99	73	73.7	65.1–82.4	0.82
On TETA monotherapy [37]	109	90	82.6	75.4–89.7	0.73
On zinc monotherapy [16]	88	63	71.6	62.2–81.0	0.84

*TBs* treatment blocks, *CI* confidence interval, *RR* relative risk ratio, *DPA* penicillamine, *TETA* trientine

**Fig. 5:**
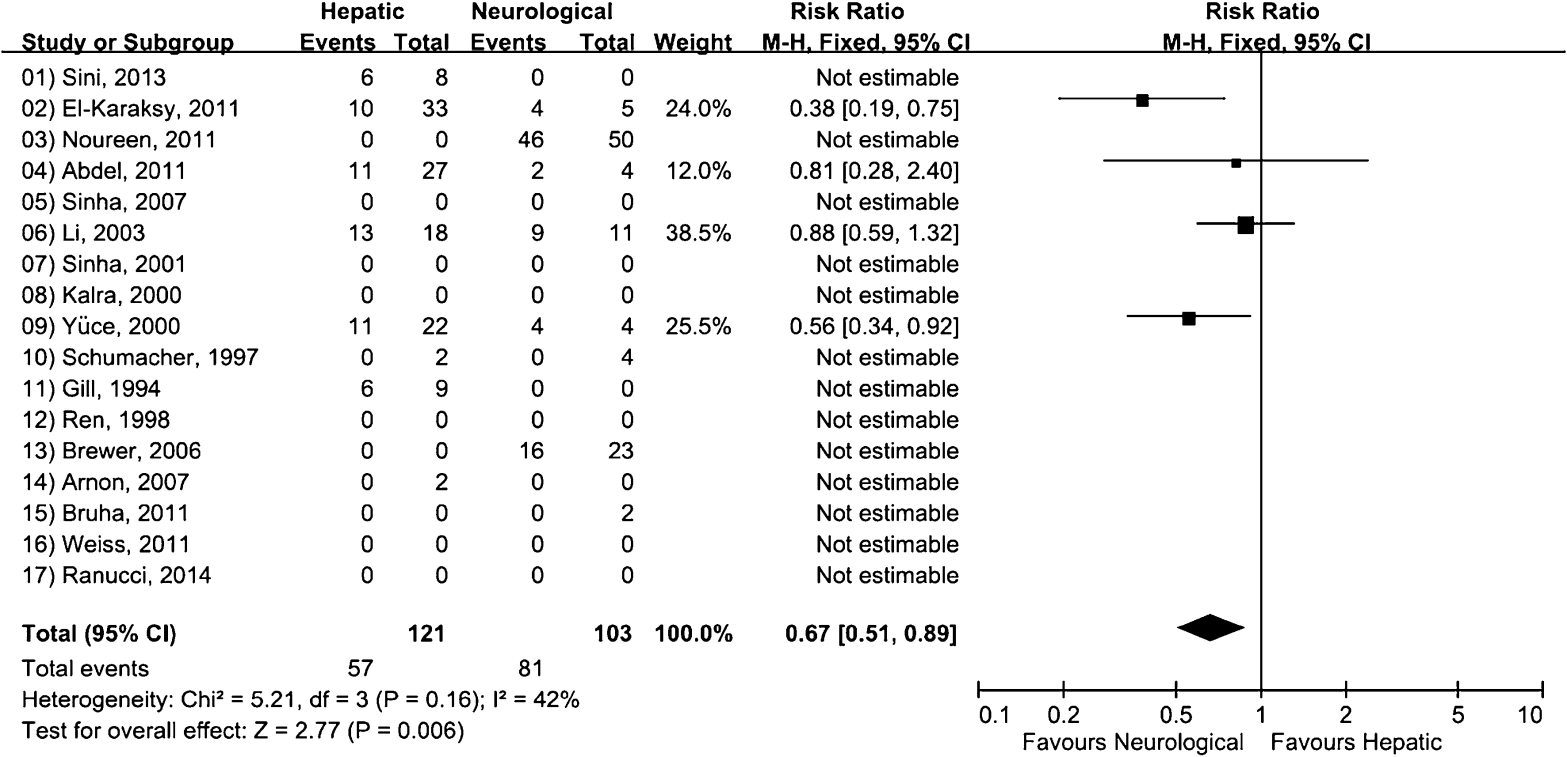
Forest plot of studies on combination therapies for hepatic phenotype versus neurological phenotype examining relative risk of effectiveness

We then searched for relationships between phenotype and combination therapy effectiveness, and found that less than one half (47.1 %, 95 % CI 38.2–56.0 %) of the TBs in the hepatic group (mixed phenotype excluded) responded well to combination therapy, compared to 78.6 % (95 % CI 70.7–86.6 %) of TBs in the neurological group (Table 2). According to our subgroup analyses (two-phenotype stratification), a statistically significant difference exists between the two subgroups (*p* = 0.02) (Fig. 5). The RR of the overall effectiveness rate was 0.63 (95 % CI 0.43–0.94), indicating that the combination therapies were 31.5 % (95 % CI 18.8–44.3 %) less effective for the hepatic patients than for the neurological patients. Note that the total number of TBs involving patients in different phenotype groups does not equal the overall effectiveness number since some of the studies in the sample did not give specific statistics for different phenotypes. A comparison of all phenotypes and combination therapy effectiveness revealed statistically significant correlations between the two factors (χ^2^(3) = 26.666, *p* < 0.001) (data not shown); medium–low positive correlations between the two variables were noted in the form of Cramer’s V value (0.321, significant at 0.001). In contrast, results from correlation and difference tests involving various combination therapy types and overall effectiveness were not statistically significant (χ^2^(1) = 0.373, *p* = 0.541 and Z = −0.611, *p* = 0.271). In other words, the data indicate that similar results are produced by all of the combination therapy types reviewed for this paper.

### Adverse Effects

**Fig. 6:**
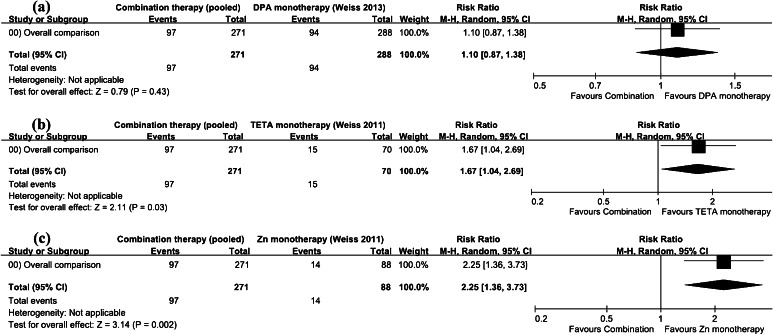
Forest plot of 17 included studies measuring relative risk of pooled adverse effects following combination therapy compared to **a** DPA, **b** TETA, and **c** Zn monotherapies

Data on combination therapy safety, including adverse effects and mortality, are presented in Table 4. Since the first combination therapy type was clearly the most common, we collapsed the other six to create a workable balance between sample sizes. Note that we split the statistics for one study [11] into two parts because the patients were treated with two different combination therapies. Since some of the studies in the sample did not specifically describe adverse reactions for different phenotypes, the numbers of TBs for different phenotypes and for overall adverse effects are not equal. Of the 271 TBs listed in Table 4, 97 resulted in adverse reactions, an overall adverse effect rate of 35.8 % (95 % CI 30.1–41.5 %). The percentage for patients in the hepatic category was 41.7 % (95 % CI 31.1–52.2 %) and that for those in the neurological category was 26.3 % (95 % CI 12.3–40.3 %), not significantly different (*p* = 0.84), perhaps due to the small sample size. Results from our analysis of inter-studies on adverse effect rates are presented in Fig. 6, with the number of events noted as the number of TBs presenting adverse effects. The data indicate that the combination therapies resulted in greater relative risk compared to those for the TETA (RR: 1.67, 95 % CI 1.04–2.69) and Zn (RR: 2.25, 95 % CI 1.36–3.73) monotherapies [16], but not that for the DPA monotherapy [37] (RR: 1.10, 95 % CI 0.87–1.38). Statistically significant differences were not noted for correlation and difference measures between different combination therapy types and overall adverse effects (χ^2^(1) = 0.938, *p* = 0.333 and Z = −0.968, *p* = 0.166).

**Table 4:** Safety investigation data for combination therapies in analyzed studies

PMID	Comb. type	Phenotype								Overall adverse effects		Mortality on combination therapy			
		Neurological		Hepatic		Mixed		Asymptomatic							
		NA+	NA−	HA+	HA−	MA+	MA−	AA+	AA−	A+	A−	Dead	LT	Alive	Mortality (%)
23011036	a											0	0	13	0.0
22055589	a	1	4	10	23	1	2			12	29	8	3	30	26.8
22355993	a											0	0	50	0.0
21682854	a	4	0	18	9	7	1	3	16	32	26	16	0	42	27.6
17709362	a											0	0	50	0.0
14759316	a									4	14	5	0	13	27.8
11837754	a											–	–	–	–
10869138	a											1	0	19	5.0
10745386	a	0	4	7	15					7	19	4	3	19	26.9
9193846	a											0	6	0	100.0
8076990	a											1	0	5	16.7
Subtotal		5	8	35	47	8	3	3	16	55	88	35	12	241	16.3
Percentage (%)		38.5	61.5	42.7	57.3	72.7	27.3	15.8	84.2	38.5	61.5	12.2	4.2	83.7	
8076990	c											1	0	2	33.3
11819363	b									22	38	0	0	60	0.0
16606763	e	5	18							5	18	4	0	19	17.4
17460493	d			0	2					0	2	0	0	2	0.0
20958917	b	0	2							0	2	–	–	–	–
21185835	f									10	20	0	1	29	3.3
24661374	b									5	6	0	0	11	0.0
Subtotal		5	20	0	2	0	0	0	0	42	86	5	1	123	4.7
Percentage (%)		20.0	80.0	0.0	100	–	–	–	–	32.8	67.2	3.9	0.8	95.3	
Total		10	28	35	49	8	3	3	16	97	174	40	13	364	12.7
Percentage (%)		26.3	73.7	41.7	58.3	72.7	27.3	15.8	84.2	35.8	64.2	9.6	3.1	87.3	

*Comb. type* combination therapy type, *A+* adverse effect, *A−* non-adverse effect, – not available, *LT* liver transplantation, *Alive* LT excluded, *Mortality* (Dead + LT)/(Dead + LT + Alive)

### Mortality

**Fig. 7:**
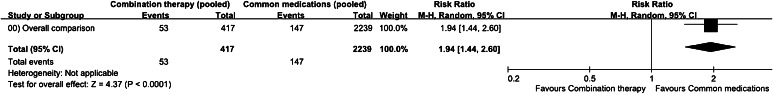
Forest plot of 17 included studies measuring relative risk of pooled mortality following combination therapy compared to pooled mortality of four most commonly used medications

Detailed mortality data associated with combination therapy studies are presented along the right-hand side of Table 4. Pooled results from mortality investigations of the four most commonly used medications (DPA, TETA and Zn monotherapies, and combination therapy) and from studies of the combination therapies alone are presented in Table 5. As shown, of the 2239 patients mentioned in 44 of the 50 studies of the four most common medications, 103 died and 44 received liver transplants, a mortality rate of 6.6 % (95 % CI 5.5–7.6 %). In contrast, the mortality rate for all patients receiving some form of combination therapy was 12.7 % (95 % CI 9.5–15.9 %), suggesting that those therapies failed to help a large number of individuals with acute WD. For this part of our analysis, we used the number of deceased and liver transplant patients as the number of events. A statistically significant difference was found between mortality rates for patients receiving combination therapies and those receiving common medications (Fig. 7; RR: 1.94, *p* < 0.001). Since we did not measure the percentages of patients who experienced acute liver failure, the two group analyses may suffer from bias. Still, the pooled data suggest that combination therapy patients had a much higher mortality rate compared to those receiving the other frequently used medications.

**Table 5:** Mortality statistics for different patient groups in included studies

No. of patients (%)	Dead	LT	Alive	Mortality (%)	Patients in total	Studies included
On common medications (pooled)	103 (4.6 %)	44 (2.0 %)	2092 (93.4 %)	6.6	2239	44 of 50
On combination therapies (pooled)	40 (9.6 %)	13 (3.1 %)	364 (87.3 %)	12.7	417	15 of 17

*LT* liver transplantation, *Mortality* (Dead + LT)/(Patients in total)

Mortality rates for patients in different phenotype groups were difficult to determine due to the small sample size and lack of mortality data for each group. However, we did find statistically significant differences in mortality among patients receiving different types of combination therapies (*p* < 0.001). Patients in the DPA + Zn sulfate group had a much higher mortality rate compared to those in all other groups (16.3 vs. 4.7 %; RR: 3.51, 95 % CI 1.54–8.00). Chi square test results indicate a statistically significant correlation (χ^2^(1) = 10.933; *p* = 0.001) between combination therapy type and mortality.

## Discussion

In light of the rarity of WD cases and lack of clinical consensus on the best medications, our goal was to systematically review the literature and to perform statistical analyses to support or refute assertions of the success of various combination therapies. Our results indicate a success rate for combination therapies of approximately 60 %, much lower than expected. That figure is significantly less than those reported by Bruha et al. for DPA monotherapy (73.7 %) [15], Weiss et al. for TETA monotherapy (82.6 %) [37], and Weiss et al. for zinc monotherapy (71.6 %) [16]. We found strong evidence indicating that hepatic patients do not respond well to combination therapies, with a reported effectiveness rate of 47.1 versus 78.6 % for patients with neurological manifestations. Results from a pooled analysis show that compared to hepatic manifestation patients, neurological patients were significantly more likely to receive benefits from combination therapies. For example, Pellecchia et al. [38] found that DPA combined with zinc is effective and safe for neurologically impaired patients. In terms of safety, the studies we reviewed reported a 35.8 % pooled adverse effect for all patients receiving some type of combination therapy (41.7 % for hepatic patients and 26.3 % for patients with neurological presentations). The lack of a statistically significant difference between the two phenotypes is likely due to the small sample size, yet there is still potential for clinical significance. Regarding mortality associated with combination therapies, the 12.7 % rate was significantly higher than the 6.6 % rate reported for common medication therapies. It is likely that the mortality rate is higher for hepatic patients, but the reviewed studies did not contain specific mortality data for that group. One unexpected finding was the higher mortality rate for patients receiving DPA plus zinc sulfate compared to other types of combination therapies (16.3 vs. 4.7 %). Thus, Yonetani and Walshe [34] emphasize the danger of using zinc sulfate with any chelation regimen.

From our analysis, it appears that the literature lacks rigorously designed studies and safety data on combination therapies using zinc and a chelator. The three most frequently cited studies that suggest favorable outcomes for combination therapies involving zinc plus either DPA or TETA are those by Dhawan et al. [30], Askari et al. [31], and Santos Silva et al. [32]. Dhawan et al.’s research focus was the scoring system for WD liver transplants [30], but in their report they claim that 20 symptomatic, non-deceased, and non-liver-transplant-receiving children did not require transplants for a long period of time after receiving a combination of DPA plus zinc. Their study is lacking in several respects: it does not include detailed evaluations regarding the clinical effectiveness of combination therapy, nor do they provide follow-up information for seven asymptomatic siblings who were treated with a combination of DPA and zinc. In their paper, Askari et al. [31] described the successful use of TETA plus zinc in eight patients with decompensated hepatic WD, but their approach involved the use of that combination therapy for 4 months, followed by a regimen of zinc monotherapy. They claim that the combination therapy reduced or eliminated the need for liver transplants, but the time period involved was imprecise. Santos Silva et al. [32] evaluated the effectiveness of DPA plus zinc for treatment periods ranging from one to 2 years, but some of the patients in their study had to be shifted to other therapies due to the adverse effects of the initial combination therapy. They mention three combination therapy patients during an initial follow-up period and four during a second follow-up period, but they are unclear about overlaps.

Some researchers [33, 34] have argued that there is no advantage to the concomitant administration of DPA and zinc, suggesting that zinc may interact with both DPA and TETA, and possibly inhibit chelator absorption and action [33, 39]. A third research team has made the strong recommendation that zinc sulfate should never be used with chelation medication [34]. Friedman and Yarze [40] also argue that it is counterproductive to use a combination of chelators and zinc in WD patients. According to EASL guidelines [19], there are no known advantages to using combination therapies involving a chelator and zinc, though they do not deny the possibility. AASLD guidelines [20] are unclear on this question, simply stating a need for more confirmatory research.

In one retrospective cohort study [16], six combination therapy TBs were discontinued because the physician suspected that the zinc and chelator were interacting pharmacologically. Arnon et al. [14] reported that two patients taking TETA monotherapy alone for 6 months had their hepatic alanine aminotransferase (ALT) levels return to normal. The decision was made to switch to a combination of TETA plus zinc, but after another 6 months their ALT levels nearly doubled, and after a full year they were almost three times the level considered normal [14]. The authors speculated that the patients may not have been adherent, but this idea was neither tested nor verified. They did, however, suggest that future combination therapy was unnecessary.

The literature contains other evidence concerning chelator-zinc interaction. In their study of urinary copper excretion following TETA monotherapy, Dubois et al. [41] reported that urinary zinc content increased from 181 μg/day pre-treatment to 402 μg/day post-treatment. Their observations were similar to those reported by McCall et al. [42] in a metabolic study involving DPA trials. Kodama et al. [43] reported a significant increase in the urinary excretion of zinc in a group of healthy (non-WD) volunteers during the first 6 hours following TETA administration. Kuchinskas and Rosen [39] investigated the affinities of bivalent metals for DPA, and reported a high-to-low affinity order of Hg > Ni > Cu > Zn > Cd > Pb; this serves as indirect evidence that DPA is capable of chelating both copper and zinc. Cossack and Bouquet [44] have described a sub-clinical deficiency of zinc induced by DPA treatment. In an animal study [45], Fieten et al. evaluated hepatic copper and zinc concentrations before and after DPA monotherapy treatment in 42 Labrador Retrievers, and reported significant decreases in both concentrations in the dogs’ livers. Combined, these studies suggest that zinc should not be combined with a chelator, even several hours apart, because doing so is likely to reduce the effectiveness of zinc for treating WD. Further, the existing evidence indicates that the presence of zinc in the bloodstream and gut may alter the effect of chelators on copper.

## Conclusion

Our main findings are (a) an overall effectiveness rate of only 47.1 % and (b) an overall adverse effect rate of 41.7 % among hepatic patients treated with combination therapies. We also found that the overall mortality rate for patients receiving a combination therapy was 12.7 %, double that reported for patients receiving the four most commonly used medications. Another important finding is that the combination therapy of DPA plus zinc sulfate resulted in much higher mortality rates compared to those for all other combination therapy types (16.3 vs. 4.7 %). However, the pooled data cannot be considered high-quality evidence for estimating the effectiveness and safety of combination therapies. Thus, these findings should be used to support treatment decisions only until more and higher-quality evidence becomes available. More large-cohort randomized clinical trials and/or evidence-based studies are still required to fully address the issues mentioned in this review. Our primary conclusion is that clinicians should closely monitor biochemical test results and clinical courses for WD patients receiving combination therapies, especially in response to hepatic manifestations.

## Limitations

Possible limitations to our findings include a lack of stratification for mild, moderate, and severe adverse effects, plus the apparent lack of high-quality evidence in support of estimates of relative effectiveness and adverse effects of combination therapies versus monotherapies. Further, there may be bias in some interpretations of results due to the lack of substantial data and additional reports on combination therapies. For these reasons, no firm recommendations can be drawn from the pooled data. Note also that WD is an intractable disease, meaning that individual patients may have different responses to each of the four most commonly used medications due to variance in WD genotypes and phenotypes. Consequently, neither a standard treatment regimen nor a clear consensus exists regarding an optimal medication therapy for treating the disease. More evidence-based studies and/or large-cohort randomized controlled comparative trials are required.
